# Environmental monitoring of waterborne *Campylobacter:* evaluation of the Australian standard and a hybrid extraction-free MPN-PCR method

**DOI:** 10.3389/fmicb.2015.00074

**Published:** 2015-02-09

**Authors:** Rebekah Henry, Christelle Schang, Gayani I. Chandrasena, Ana Deletic, Mark Edmunds, Dusan Jovanovic, Peter Kolotelo, Jonathan Schmidt, Richard Williamson, David McCarthy

**Affiliations:** ^1^Environmental and Public Health Laboratory, Department of Civil Engineering, Monash UniversityClayton, VIC, Australia; ^2^ALS EnvironmentalScoresby, VIC, Australia

**Keywords:** *Campylobacter*, PCR, estuary, inter-laboratory, environmental interactions, culture

## Abstract

*Campylobacter* is the leading agent of diarrheal disease worldwide. This study evaluates a novel culture-PCR hybrid (MPN-PCR) assay for the rapid enumeration of *Campylobacter spp*. from estuarine and wastewater systems. To first evaluate the current, culture-based, Australian standard, an inter-laboratory study was conducted on 69 subsampled water samples. The proposed Most-Probable Number (MPN)-PCR method was then evaluated, by analysing 147 estuarine samples collected over a 2 year period. Data for 14 different biological, hydrological and climatic parameters were also collated to identify pathogen-environment relationships and assess the potential for method specific bias. The results demonstrated that the intra-laboratory performance of the MPN-PCR was superior to that of AS/NZS (σ = 0.7912, *P* < 0.001; κ = 0.701, *P* < 0.001) with an overall diagnostic accuracy of ~94%. Furthermore, the analysis of both MPN-PCR and AS/NZS identified the potential for the introduction of method specific bias during assessment of the effects of environmental parameters on *Campylobacter spp*. numbers.

## Introduction

Campylobacteriosis is a zoonosis spread into the environment through the release of fecal material. Current WHO figures suggest that *Campylobacter* are the leading cause of diarrheal disease in industrialized nations with annually more than 60, 000 and 17, 000 confirmed cases reported respectively in the United Kingdom (UK) and Australia alone (Corvisy, 2013; Hughes and Gorton, 2013). The primary route of infection is through ingestion of contaminated food products. However, environmental sources, such as water used for recreational purposes and stormwater flows, represent an often overlooked source of disease transmission (Adak et al., 1995; Pond, 2005; Arnone and Walling, 2007); 3% of confirmed cases in the UK were reported as the direct result of contact with contaminated water supplies (Anonymous, 2000). *Campylobacter* survival within non-biological settings (i.e., water and soils) (Thomas et al., 1999; Ross and Donnison, 2006; Donnison and Ross, 2009; Rodríguez and Araujo, 2012), is dependent on numerous exogenous variables. Sensitivities to seasonal variations, temperature, sunlight exposure and dissolved nutrients have been observed to directly affect concentrations of the bacterium within water sources (Jones, 2001; Boyle et al., 2008; Maal-Bared et al., 2012; Rodríguez and Araujo, 2012). Thus, variations in climatic, biological and hydrological conditions have direct implications on human health outcomes (Patz et al., 2003).

Enumeration of *Campylobacter* from complex source samples can be difficult due to the fastidiousness and fragility of the organism (Pitkänen, 2013). Furthermore, isolation from urban waters is problematic, as they are usually present at low concentrations (Koenraad et al., 1997). Culture-based methods for the enumeration and isolation of *Campylobacter* from waters have become the international standard (Standardization ISO, 2005). The addition of concentration and pre-enrichment techniques and application of selective media has significantly improved recovery efficiencies (AS/NZS, 2001; Jokinen et al., 2012; Ugarte-Ruiz et al., 2012). However, culture-based methods are time-consuming and expensive, requiring filtration, selective enrichment, isolation and biochemical confirmation (~9 days to report).

The application of molecular tools, such as PCR, may help to circumvent some of the limitations of current methods. Assays for the detection of *Campylobacter* have been trialed and the results found to be comparable to culture-based methods (Savill et al., 2001; St-Pierre et al., 2009). It is important to note that the majority of assays were conducted on food products, primarily chicken rinses, with a limited number of environmental studies (Pitkänen, 2013). However, despite observed between-technique correlations, only three ISO methods currently utilize PCR for the detection of bacterial pathogens (Ireland NSAo, 2012; Organisation IS, 2012; Standardization ISO, 2013). One possible explanation for the lack of up-take of these methods, in water studies, is the large volume of water that needs to be filtered in order to detect low concentration microbes. Consequently, exogenous variables, such as humic acid (a principle organic component of soil and known PCR inhibitor (Schrader et al., 2012), are also concentrated (Lübeck et al., 2003). The ability of laboratories to remove or limit humics, and other inhibitory substances, within DNA samples may introduce inter-laboratory variability in reporting. However, with the globalization of molecular tools, such as DNA purification kits and PCR master-mixes, the variations between laboratories can be minimized and should be no different to those observed for culture-based techniques.

A further consideration is the limited ability of researchers to remove exogenous naked DNA and DNA derived from non-viable cells. Direct amplification of environmental samples can result in the over-estimation of risk if the presence of free DNA is not accounted for. The use of chemical pre-treatments, such as propidium monoazide (PMA), has been proposed for the selective removal of free and non-viable cell DNA (Nocker et al., 2006, 2007). However, the efficiency of these methods to completely remove DNA from non-viable *Campylobacter* is still under investigation (Pacholewicz et al., 2013). Prior enrichment of samples, by culture based techniques, has been demonstrated to promote detection of viable cells while limiting the presence of exogenous DNA (Abulreesh et al., 2006).

Alternative hybrid methods employing cultural enrichment and PCR confirmation to enumerate *Campylobacter* in environmental samples have been described (Savill et al., 2001; Sails et al., 2003; Nam et al., 2005; St-Pierre et al., 2009; Rodriguez and Araujo, 2010). The assays have been successfully applied to complex matrices including feces, soil, foodstuffs and some recreational waters (Hernandez et al., 1995; Savill et al., 2001; Kulkarni et al., 2002; Josefsen et al., 2004a; Khan et al., 2009; St-Pierre et al., 2009; Rodriguez and Araujo, 2010; Rodgers et al., 2012; Gharst et al., 2013; Rohonczy et al., 2013; Taboada et al., 2013), demonstrating their broad application potential. The procedures utilize the benefits of standard filtration and culture to isolate organisms in combination with PCR-assays for rapid sensitive detection. The advantage of applying such procedures is that the presence of inhibitory substances from concentrated samples can be limited or diluted to enable reproducible assay results. Furthermore, initial culture-based enrichment increases the number of viable cells for later PCR amplification procedures. However, current hybrid protocols remain overly complicated often requiring multiple enrichment steps, centrifugation and specialized DNA purification procedures (Savill et al., 2001; Sails et al., 2003; Nam et al., 2005; St-Pierre et al., 2009; Rodriguez and Araujo, 2010; Pitkänen, 2013; Rohonczy et al., 2013). For universal uptake, a successful standard procedure should require minimal specialized equipment and resources, be easily applied with good correlation across laboratories and short reporting time.

The complexity and interaction of variables within estuarine and stormwater systems has limited the use of direct culture and molecular-based methods for *Campylobacter* enumeration (Lampard et al., 2012). However, hybrid methods have not been tested directly on these systems. Here we describe and evaluate a novel, DNA-purification free, culture-PCR hybrid assay for the rapid detection and enumeration of pathogenic *Campylobacter* from estuarine and wastewater systems. Concurrently, an inter-laboratory study was conducted to evaluate the diagnostic accuracy of the current standard, AS/NZS 4276.19:2001 (AS/NZS). AS/NZS is a MPN culture-based method requiring filtration of complex samples prior to cultivation and biochemical confirmation of bacterial genus. The study encompassed 147 samples collected over a 2 year period to evaluate the potential of the MPN-PCR method as a standard *Campylobacter* enumeration procedure for environmental waters. Environmental parameter relationships, which significantly affect *Campylobacter* concentrations and assessment of risk and human health outcomes, were also evaluated for method specific bias.

## Materials and methods

### Study locations and sample collection

Samples were collected from two systems in Victoria, Australia: the Yarra River and Monash University stormwater harvesting system.

**Figure 1:**
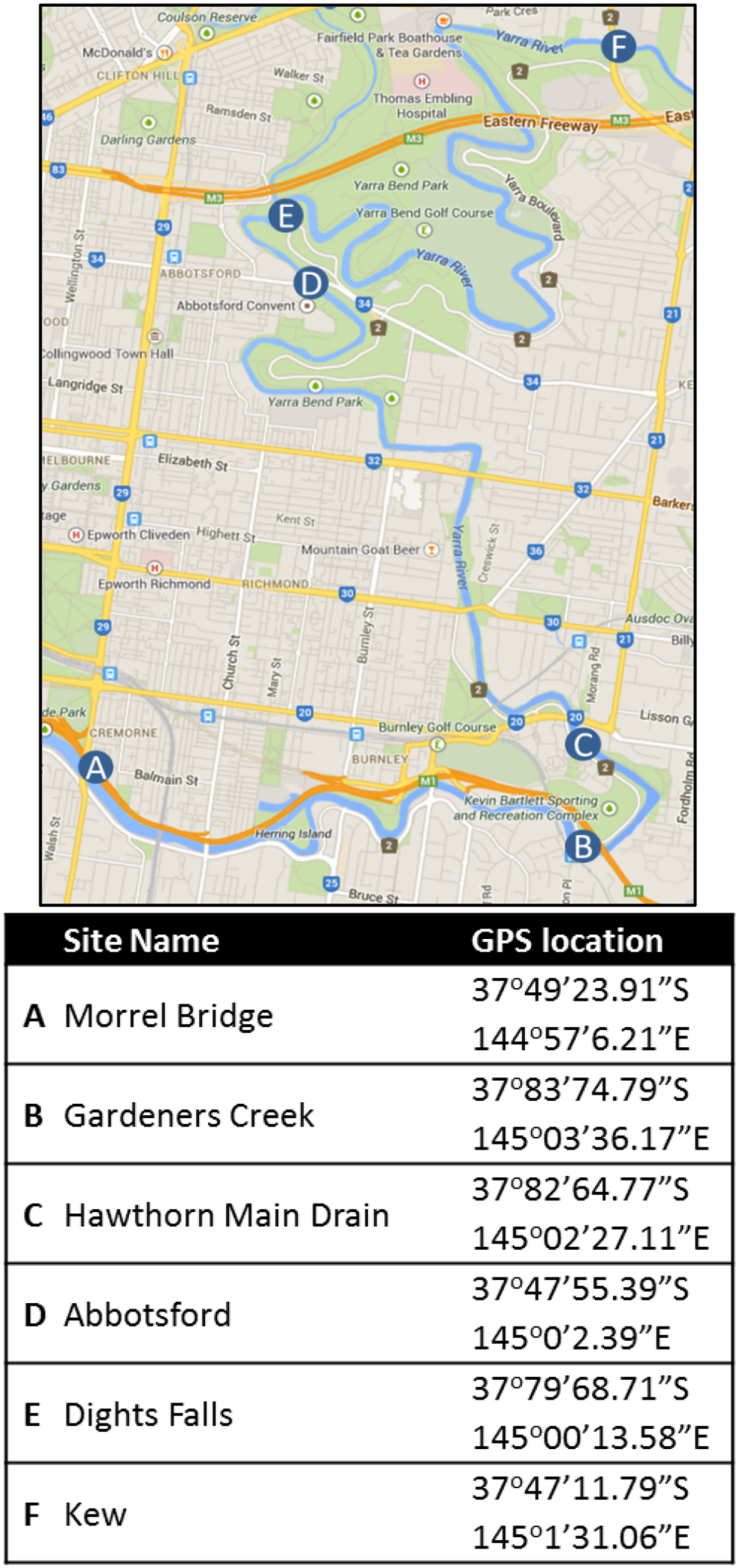
**Location and GPS co-ordinates of the study sites in the Yarra River, Melbourne**. Sampling locations within the Yarra River; estuarine; Morell Bridge (A), Hawthorn Main Drain and (C) Abbotsford (D). Outside of the estuary, Dights Falls (E) and Kew (F) are located in the fresh water reach and Gardiners Creek (B) is an urban creek input. Map sourced from Google Maps (https://www.google.com.au/maps/).

Five sampling sites from within the Yarra River estuary (Melbourne, Australia) were selected for study from January 2012 to December 2013 (Figure 1). These consisted of two estuarine locations [Abbotsford (Abts) and Morrel Bridge (Mor)], two fresh water inputs (Kew and Dights Falls) and two urban stormwater inputs, Gardiners Ck (Gard) and Hawthorn Main stormwater drain (HMDE). Sites were selected to enable measurement of *Campylobacter* concentrations in source waters within the boundaries of the estuary. The water column within the estuary ranges from completely fresh at the riverine end (Kew/Dights Falls; 0.06–0.13 psu 5th, 95th percentile) to a salt water region at the seawater boundary. The Abbotsford site at the beginning of the estuarine section of the Yarra River was predominantly fresh (0.06–0.15 psu; 5th; 95th percentile), while Morell Bridge exhibited strong stratification driven by salt wedge (top layer salinity 0.73–9.25 psu; bottom layer 3.78–28.68 psu). The geographical positions and location descriptions for each site are presented in Table 1. Estuarine water grab samples were taken 3 m perpendicular from the bank and at an approximate depth of 0.15 m at each location. Samples were collected into 2 L polyethylene terephthalate containers that had been rinsed with a minimum of 1 L of source water prior to sample collection. The dates on which each sampling was undertaken are specified in Supplementary Material. A total of 147 estuarine samples comprising, 42 Abts, 45 Mor, 13 Kew, 16 Dights Falls, 34 Gard and 6 HMDE, were collected. Sampling days were selected to incorporate variable climatic and hydrological conditions. Rain event samples were collected using a flow-weighted strategy (McCarthy et al., 2008).

**Table 1:** **Sampling site description**.

	**Site name**	**Description**
A	Morrel bridge	High density urban developments; high watercraft usage; Queens Bridge Drain located around 950 m upstream; deep, wide channel of salt and fresh water
B	Gardeners creek	Completely channelized section of Gardiners Creek; extremely receptive to rainfall events within the catchment; No tidal effect; High density industrial and residential areas upstream of site; no watercraft activity; surrounded by recreational/sporting grounds
C	Hawthorn main drain	Major urban stormwater drain. Collects stormwater inputs from high density industrial and residential areas; no watercraft activity
D	Abbotsford	Shallow fast-flowing riffled section; high density industrial and residential developments with recreational parklands; predominantly fresh water
E	Dights falls	Site ~20 m upstream of weir; surrounded by parklands; no tidal influence; minimal watercraft; Merri creek junction just upstream; Eastern Freeway crosses Merri Creek just U/S of Merri-Yarra junction
F	Kew	Low density industrial, medium level residential developments; minimal watercraft activity; no tidal affect; fresh water

Water samples derived from a stormwater biofiltration system were included as part of the inter-laboratory evaluation of AS/NZS. The Monash University biofilters are located in Clayton, Australia, and treat stormwater from a 4500 m^2^ multi-story carpark. The stormwater is initially fed through large basins which allow some sediment to settle (Hatt et al., 2009; Chandrasena et al., 2012). The biological filters are planted *Carex apressa* and *Melaleuca ericifolia*. As the stormwater moves through the sand-based media, the pollutants (nutrients, microbes and heavy metals) are removed through physical, chemical and biological processes. Six 30 L low complexity outflow samples, containing an estimated <10 MPN/L *C. jejuni* NCTC 11168 were collected. At the outlet, an electromagnetic flow meter (Magflow by SIEMENS) was connected to monitor flow rate. The data was stored in a Campbell CR200 data logger which triggered a Sigma 900 autosampler every 10,000 L. A Teflon sampling tube was inserted into the outlet pipe from which ten 3 L sub samples were collected into clean polyethylene terephthalate containers.

All samples were placed on ice, divided into replicate samples and delivered to (1) Monash University, Environmental and Public Health Laboratory (Lab-Res) and (2) ALS Environmental (Lab-Comm). ALS is a facility accredited by the National Association of Testing Authorities (NATA). Delivery and analysis occurred between 4 and 6 h of initial sample collection. Samples that underwent inter-laboratory evaluation of AS/NZS are outlined in Supplementary Material.

### Culture-based multi-tube analysis of *Campylobacter* spp. in water samples

To calculate the inter-laboratory reproducibility of AS/NZS the procedure was conducted at two independent laboratories. Lab-Res was less than 1 h travel time from Lab-Comm; this small travel time was essential to prevent significant changes in the microbial content of samples, and the introduction of unwanted biases.

**Figure 2:**
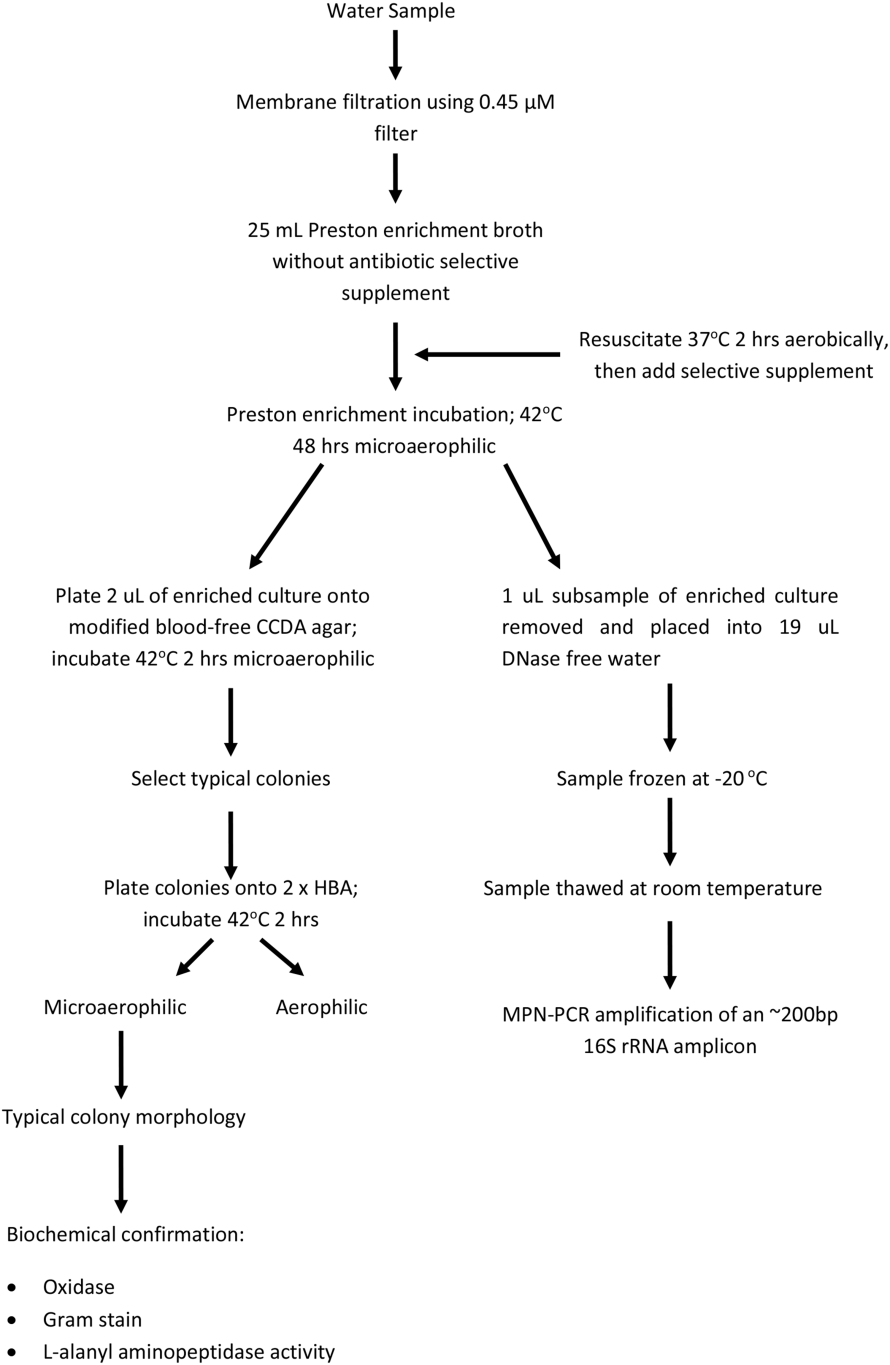
**Flow chart for the isolation and confirmation of *Campylobacter spp*. following membrane filtration of water samples**. Adapted from AS/NZS 4276.19:2001 (AS/NZS, 2001).

All samples underwent membrane filtration and examination for thermophilic *Campylobacter spp*. as described in the Australian/New Zealand Standard 4276.19:2001 (AS/NZS, 2001) (outlined in Figure 2) with the following modifications. Five or eleven tube MPN analyses were conducted, dependent on sample source and whether the *Campylobacter* concentrations were expected to be high. The number of tubes per sample, and the volumes filtered for each tube, are listed in Supplementary Material. Both laboratories used equivalent filtration volumes and number of tubes for each sample. For 11 tube MPN tests, two main filtrate regimes were applied: (1) 2 × 250, 3 × 100, 3 × 50, and 3 × 10 mL (2) 1 × 500, 5 × 100, and 5 × 10 mL. For 5 MPN tube tests, three main filtrate regimes were used: (1) 50, 15, 5, 1.5, and 0.5 mL, (2) 250, 100, 50, and 2 × 1 mL (3) 500, 250, 100, and 2 × 10 mL. Post-filtration onto 0.45 μM cellulose nitrate filters (Sartorius, Germany) samples were placed into 25 mL Prestons broth and resuscitated aerobically for 2 h at 37°C. *Campylobacter* selective supplement (Oxoid, United Kingdom) was added as per manufactures instructions and broths enriched for 48 h at 42°C. As outlined in AS/NZS, two 25 mL broth cultures were spiked with *Escherichia coli* strain ATCC 11775 or *C. jejuni* NCTC 11168 as negative and positive reaction controls respectively. To ensure no post-collection environmental contamination, DNA-free water, equivalent to the highest filtrate volume, was left opened to the environment for the duration of filtration and then filtered onto a 0.45 μM filter, placed into Prestons broth and enriched as described in AS/NZS. No antibiotic negative enrichment controls were included to ensure no media contamination. *Campylobacter jejuni, E. coli*, no antibiotic and DNA-free water contamination controls were conducted with each assay at Lab-Res, while *C. jejuni* and *E. coli* controls were conducted at the commercial lab [as outlined in (AS/NZS, 2001)].

Post-enrichment (48 h at 42°C), 2 μL of each sample was plated onto modified CCDA-Preston and incubated for 48 h at 42°C (Oxoid, United Kingdom). Typical colonies were selected based on comparison to the positive control strain, and plated, in duplicate, onto Horse Blood Agar (HBA) (Oxoid, United Kingdom). One of each of the HBA plates were incubated under either aerophilic or microaerophilic conditions for 48 h at 42°C after which biochemical confirmation of *Campylobacter* using the Oxoid Biochemical Identification System (O.B.I.S) (Oxoid, United Kingdom) was conducted on isolates present under microaerophilic conditions (Figure 2).

### MPN-PCR analysis of *Campylobacter* spp. from enriched environmental isolates

Post-enrichment cultures, described above, were removed from the incubator and plated onto modified Prestons agar (AS/NZS, 2001) (Figure 2). Concurrently, a 1 uL sub-sample was taken from each 25 mL Preston enrichment (including *Campylobacter jejuni, E. coli*, no antibiotic and DNA-free water controls) and diluted 1:20 in DNase/RNase free water and frozen at −20°C prior to use (Figure 2). These samples were freeze-thawed (one cycle) at −20°C to fracture cells prior to PCR amplification. One cycle was assumed to be sufficient to release DNA for PCR amplification. Based on the results of Lübeck et al. (2003) the forward and reverse primer pair of OT1559 (5′ CTGCTTAACACAAGTTGAGTAGG 3′) (Uyttendaele et al., 1994) and 18-1 (5′ TTCCTTAGGTACCGTCAGAA 3′) (Uyttendaele et al., 1994) were selected for specific amplification of an ~200 bp product from *C. jejuni, C. coli, C. lari* and *C. upsaliensis*. Each 12 uL reaction consisted of 5.5 μL of SSoFast Evagreen Supermix (Biorad, USA), 25 nM of each primer, 2.3 μL of DNase/RNase free water and 2 μL of lysed sample. Each sample PCR was conducted in duplicate, a no template control (NTC) was included in all assays. Amplification was performed on a Biorad CFX96 Real-Time PCR system (Biorad, USA) under the following conditions: 1 cycle of 95°C for 3 min; 40 cycles of 95°C for 5 s, 56°C for 30 s, with a plate read conducted after each cycle for fluorescence measurement. Melt curve analysis was conducted at the completion of 40 cycles of amplification and compared to the *C. jejuni* positive, NTC and *E. coli* negative controls.

### Environmental data

For Yarra River samples, rainfall (mm), temperature (°C), humidity (%) and mean sea level pressure (MSLP; hPa) data was averaged from gauge measurements taken at the Melbourne Regional Office (Station ID: 86071) and available from the Bureau of Meteorology (http://www.bom.gov.au/climate/data-services). Average daily flow rates were available at Abbottsford, Morell Bridge and Gardiners Ck. Total nitrogen (mg/L), total phosphorus (mg/L), total suspended sediment (TSS; mg/L) were measured by the Water Study Centre (Monash University, Australia) following the procedures described in APHA-AWWA-WEF (Association APH, 2005). Electric conductivity (EC; mS/cm), dissolved oxygen (DO; mg/L), turbidity (NTU) and salinity (%) were measured *in-situ* and in the river flow, when possible, using a Horiba multi-probe (HORIBA, Japan). Hydrological and environmental parameter data was not collected for biofilter derived samples.

A 10 mL subsample of the environmental water was taken and examined for the presence of fecal coliforms and *E. coli* using the Colilert® MPN method (IDEXX, USA) outlined in AS4276.21-Method 21 (Australia S, 2005). The method was conducted by Lab-Res with results presented as MPN/100 mL (Supplementary Material). A 1:10 dilution was used for all samples except when rainfall preceding collection was >3 mm where a 1:100 dilution was applied. A negative deionized water control was included for all assays.

### Statistical data analysis

The inter- and intra-laboratory relationship between AS/NZS and MPN-PCR consisted of discrete data and were therefore assessed using the Kappa coefficient of agreement (Carletta, 1996). The Kappa coefficient measures difference based on a scale from −1 to 1, where a value of 1 indicates complete agreement, 0 suggests a value has been obtained by chance and −1 a disagreement between results (Viera and Garrett, 2005). Summary statistics were also used to compare the AS/NZS results from the two independent laboratories. Wilcoxon matched-pairs signed rank test was conducted to compare paired concentration data from Lab-Res and Lab-Comm. Minimum and maximum values are summarized as well as the 5th and 95th percentile. Wilcoxon matched-pairs signed rank test was also applied to compare non-equivalent data derived from AS/NZS and MPN-PCR methods. Distribution patterns were plotted using box plots (Graphpad Prism 6.0, Graphpad Software Inc., USA) to demonstrate the reproducibility of the method.

The diagnostic sensitivity, specificity and likelihood ratios of the MPN-PCR method and AS/NZS were calculated globally and for each of the major filtration regimes. (Hoorfar and Cook, 2003; Šimundić, 2008). Information on true positive (TP MPN-PCR), false positive (FP MPN-PCR), true negative (TN MPN-PCR) and false negative (FN MPN-PCR) were collated for the MPN-PCR by comparison to AS/NZS. Equivalent information for AS/NZS was collated by comparison of inter-laboratory culture-based results. For the current study, diagnostic sensitivity (TP/TP+FP) was defined as the ability of the assay to identify a positive result when *Campylobacter* were actually present (TP) (Cook et al., 2007). Diagnostic specificity (TN/TN+FN) was defined as the discriminatory ability of the assay to identify that *Campylobacter* were absent when they was truly absent (TN) (Cook et al., 2007). The likelihood ratio (LR) was defined as the likelihood that a given result would be expected in a positive tube as opposed to a negative tube (Deeks and Altman, 2004). The more distant a LR-ratio value was from a value of one, the stronger the evidence for the presence or absence of *Campylobacter* within the sample (Deeks and Altman, 2004). Positive likelihood ratios ((sensitivity/100)/1-(specificity/100)) of ≥10 and LR- ratios ((1- (sensitivity/100)/(specificity/100)) of <0.1 were considered to provide strong evidence to rule-in/rule-out conclusions under most conditions tested (Deeks and Altman, 2004). Diagnostic accuracy (TP+TN/total sample number) was used to compare the performance of the MPN method to AS/NZS (Šimundić, 2008). Intra-laboratory evaluation of the diagnostic potential of the MPN-PCR was conducted as outlined in ISO 22174:2005 (Standarisation ISO, 2005). The standard presents the minimum requirements for PCR-based detection of bacteria within food and has been applied previously to *Campylobacter* assays (Josefsen et al., 2004a,b,c).

Statistical analysis was conducted using GraphPad Prism 6.0 (Graphpad Software Inc, USA) and SPSS Statistics 22 (IBM Statistics, USA). Spearman Rank correlation coefficients (Spearman, 2010) were conducted on Yarra River data to identify significant relationships for inter-laboratory and intra-laboratory method comparison data, within and between site (spatial) data and *Campylobacter* and data derived from the selected 14 environmental and biological parameters. The concentration differences between AS/NZS and MPN-PCR were also compared for each parameter to identify method specific bias. Biofilter data was included in correlative assessment of relationships between Lab-Res and Lab-Comm results. Due to the small sample size (*n* = 6) environmental parameter relationships were not assessed for these samples. For Spearman rank analysis, results below detection were taken as half the detection limit to allow comparative assessment as has been previously described for non-detect data (Helsel, 2004). Correlative analysis was not conducted between parameters where <10 data points were available to enable confidence interval calculation (Zar, 1999).

## Results

### Inter-laboratory method comparison

**Figure 3:**
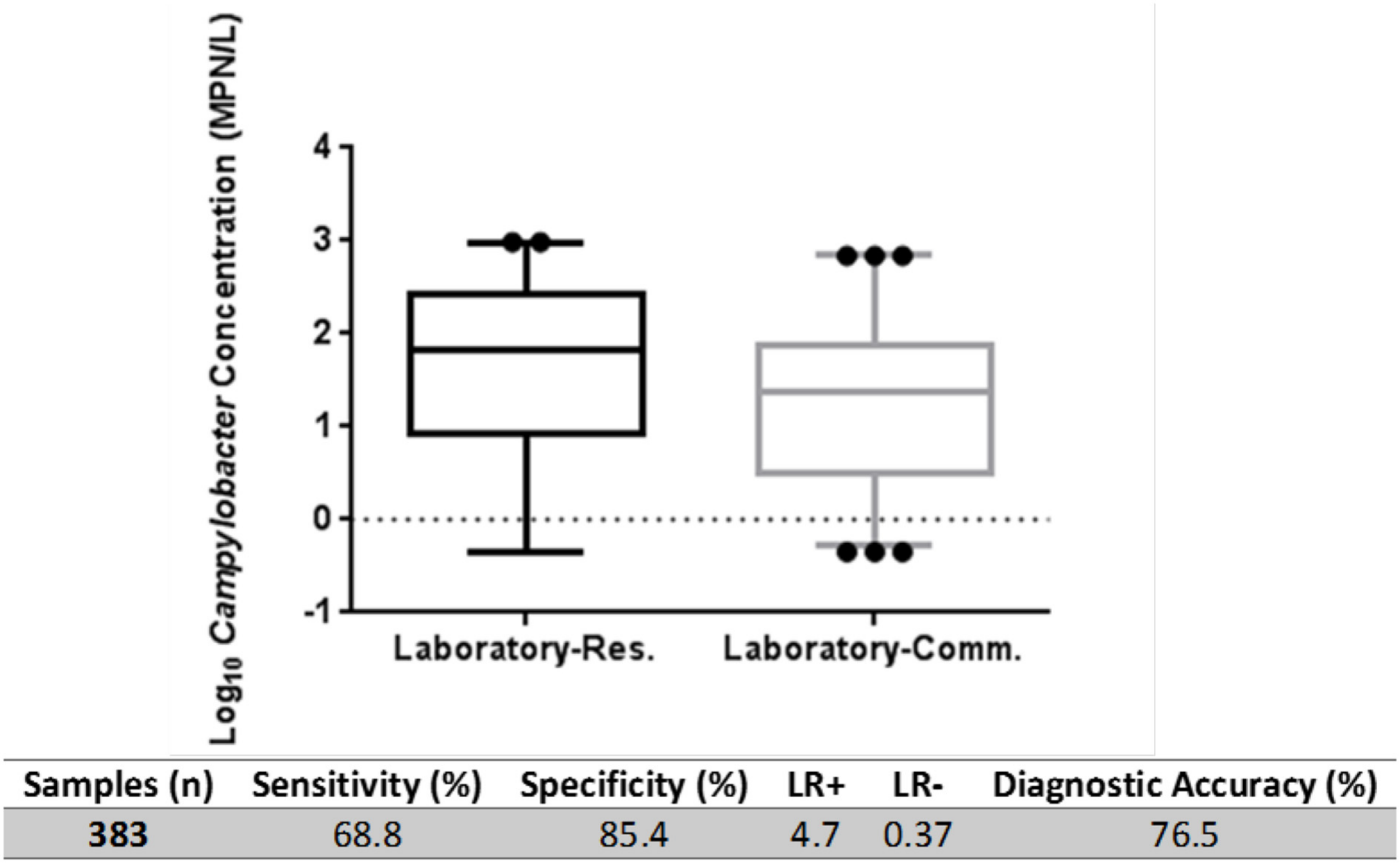
**Inter-laboratory comparison of culture-based AS/NZS method**. Box plots show median concentration of *Campylobacter spp*. derived by Laboratory-Research (Laboratory-Res.) and Laboratory-Commercial (Laboratory-Comm.) using AS/NZS 4276.19:2001. Outliers are indicated (dots). Calculation of diagnostic specificity, selectivity, LR ratio's and diagnostic accuracy as described (Hoorfar and Cook, 2003; and Šimundić, 2008). Calculations based on total assays conducted (n) irrespective of volume filtered.

**Figure 4:**
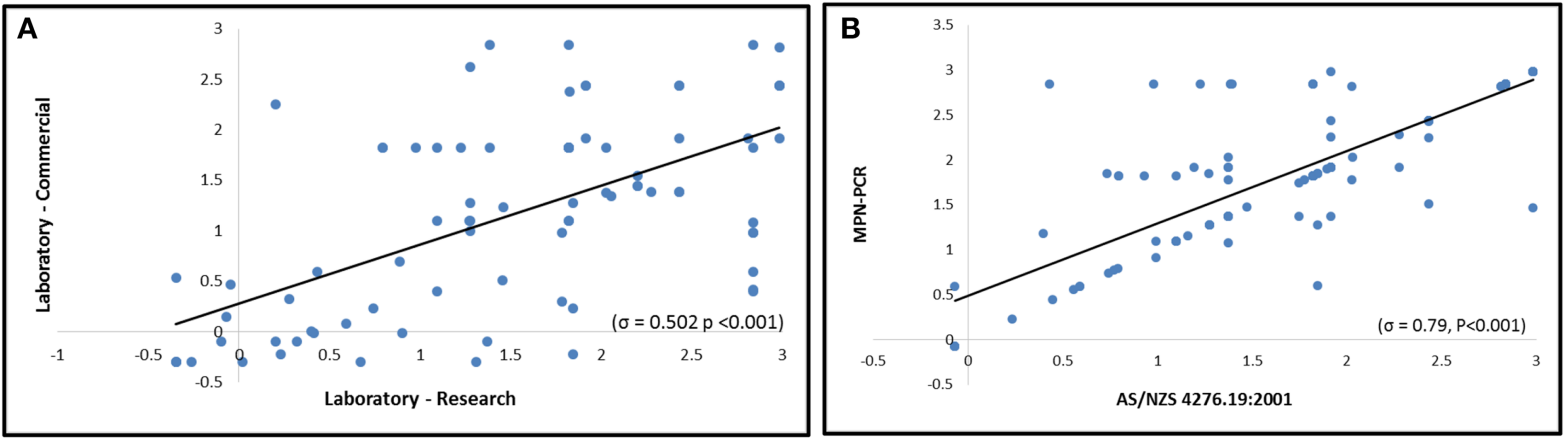
**Comparison of *Campylobacter* concentrations (Log10 MPN/L) as derived by AS/NZS and/or MPN-PCR**. **(A)** Correlative comparison of the culture based method AS/NZS by Laboratory-Research and Laboratory-Commercial (*n* = 69). **(B)** Intra-laboratory comparison of *Campylobacter* concentrations, by Laboratory-Research, using AS/NZS and MPN-PCR methods (*n* = 147).

The multi-tube AS/NZS was conducted on 69 environmental samples concurrently at two laboratories (Lab-Res and Lab-Comm see Supplementary Material for details). Summary statistics of the two datasets are presented in Figure 3. The global sensitivity and specificity of AS/NZS was assessed for all inter-laboratory investigated samples and was determined to be 68.8 and 85.4% respectively. The LR+ ratio was 4.7 and LR- ratio 0.37 with the overall diagnostic accuracy of AS/NZS observed to be 76.5%. A positive correlation (σ = 0.502, *P* < 0.001) and moderate agreement (κ = 0.531; *P* < 0.05) was observed between the results of Lab-Res and Lab-Comm (Figure 4). Lab-Res results were higher than those of Lab-Comm on 34 occasions (67%); an observation that was echoed by the significant difference found between the median concentrations of the two labs (*P* > 0.001). In all assays, the control samples generated expected results.

### Intra-laboratory comparison of AS/NZS and MPN-PCR

A total of 147 samples derived from the Yarra River estuary were analyzed concurrently by MPN-PCR and AS/NZS (Table 2). The strong positive correlation between the two methods was observed (σ = 0.7912, *P* < 0.001). Kappa coefficient results also supported significant agreement between the methods (κ = 0.701, *P* < 0.001) (Figure 4). However, in 41 samples, the MPN/L were not equivalent (MPN-PCR≠AS/NZS) (Table 2). Notably, the MPN-PCR method resulted in significantly higher detected concentrations (*P* < 0.01) of *Campylobacter spp*. within 29 of the 41 non-equivalent samples (70%) with a median concentration of 82 MPN/L whereas the median concentration for AS/NZS was 24 MPN/L. All control samples behaved as expected in both the MPN-PCR and AS/NZS.

**Table 2:** **Yarra River *Campylobacter spp*. data included in this study**.

**Site**	**All data**	**MPN-PCR=AS/NZS**	**MPN-PCR≠AS/NZS^a^**	**MPN-PCR>AS/NZS^b^**
Kew	10	7 (70%)	3 (30%)	2 (66.7%)
Dights falls	16	12 (75%)	4 (25%)	3 (75%)
Abbotsford	42	28 (66.7%)	14 (33.3%)	12 (85.7%)
Morell	45	34 (75.6%)	11 (24.4%)	8 (72.7%)
Gardiners Ck	34	25 (73.5%)	9 (26.5%)	4 (44.4%)
Total	147	106 (72.1%)	41 (27.9%)	29 (70.7%)

*Bracketed numbers represent % contribution to total samples from each investigated condition*.

a *Number of samples and percentage of samples where MPN-PCR Campylobacter concentrations (MPN/L) were not equal to (≠) that of AS/NZS*.

b *Number of samples and percentage derived from comparison of samples where MPN-PCR concentrations were greater than (>) those of AS/NZS from the MPN-PCR≠AS/NZS dataset*.

The global sensitivity and specificity of the PCR assay was assessed for the three main 5 tube MPN filtration regimes, described earlier, applied in the study (Table 3). The highest assay sensitivity (100%) was observed when 100, 50, and 1 mL filtrates were used within a single assay. The highest specificity was observed in assay volumes of 500 and 250 mL (100%), but it is important to note the relatively small number (*n* = 13) of samples investigated using this filter regime. Likelihood ratios were calculated for each filtration regime. The results indicate that the highest LR+ value, 14.2, was for assay 1 (50, 15, 5, 1.5, and 0.5 mL) while assay 2 (250, 100, 50, and 1 mL) had the lowest LR- value, 0.006. The LR values for the MPN-PCR, irrespective of filtered volume, were 9.4 (LR+) and 0.03 (LR-). The diagnostic accuracy of all regimes was high at ~94%.

**Table 3:** **Comparison of MPN-PCR method to the culture-based AS/NZS method for three 5MPN filtration regimes**.

**Vol. filtered (mL)**	**Number of samples^a^**				**Samples (n)^d^**	**MPN-PCR^b^**				
	**C+P+**	**C+P−**	**C−P+**	**C−P−**		**Diagnostic sensitivity (%)^c^**	**Diagnostic specificity (%)^c^**	**LR+^c^**	**LR−^c^**	**Diagnostic accuracy (%)^c^**
50	59	3	1	2	65	95.2	66.7	2.9	0.07	93.8
15	48	1	3	13	65	97.95	81.3	5.2	0.03	93.8
5	36	1	3	25	65	97.3	89.3	9.1	0.03	93.8
1.5	17	1	1	46	65	94.4	97.9	44.4	0.06	96.9
0.5	8	2	2	53	65	80.0	96.4	22	0.21	93.8
All	**168**	**8**	**10**	**139**	**325**	**95.5**	**93.3**	**14.2**	**0.05**	**94.5**
250	53	1	7	6	67	98.1	46.2	1.8	0.04	88.1
100	56	0	2	9	67	100.0	81.8	5.5	0	97.0
50	51	0	2	14	67	100.0	87.5	8.0	0	97.0
1 (^*^2)	21	0	8	105	134	100.0	92.9	14.1	0	94.0
All	**181**	**1**	**19**	**134**	**335**	**99.5**	**87.6**	**8.0**	**0.006**	**94.0**
500	10	1	0	2	13	90.9	100.0	n/a	0.09	92.3
250	9	1	0	3	13	90.0	100.0	n/a	0.1	92.3
100	6	1	2	4	13	85.7	66.7	2.6	0.21	76.9
10 (^*^2)	10	0	3	13	26	100.0	81.3	5.3	0	88.5
All	**35**	**3**	**5**	**22**	**65**	**92.1**	**81.5**	**4.97**	**0.09**	**87.7**
Total^d^	**384**	**12**	**34**	**295**	**725**	**96.96**	**89.7**	**9.4**	**0.03**	**93.7**

a *C+ represents culture positive, C− culture negative, P+ is MPN-PCR positive and P− represents MPN-PCR negative*.

b *Calculation of diagnostic accuracy based on comparison to the AS/NZSculture reference method of the same laboratory*.

c *Calculation of diagnostic specificity, selectivity, LR ratio's and diagnostic accuracy as described (Hoorfar and Cook, 2003; Šimundić, 2008)*.

d *Calculations based on total assays conducted irrespective of volume filtered (n)*.

*Bold values highlight the results for all samples applied to the specific filtration regime*.

* *Indicates where a sub-sample has been taken twice of the same volume*.

### Spatial relationships between *Campylobacter* spp. concentrations

Positive spatial correlations were observed between *Campylobacter spp*. concentrations at Abbotsford (D) and Dights Falls (E) (σ = 0.53, *p* < 0.05), using the AS/NZS method, and more significantly at Morell Bridge (A) and Dights Falls (σ = 0.74, *p* < 0.01) using the MPN-PCR method (Figure 1). An equivalent significant result could not be achieved using the MPN-PCR to establish a relationship between Abbotsford and Dights Falls, or applying the AS/NZS at Morell Bridge and Dights Falls. Positive spatial correlations were also established between Kew (F) and Gardiners Creek (B) (σ = 0.69, *P* < 0.05) as well as Gardiners and Morell Bridge (σ = 0.9, *P* < 0.01). However, these relationships were the result of comparisons between AS/NZS (Kew, Abbotsford) and MPN-PCR (Gardeners, Morell Bridge) and not due to the application of a single method. No other correlative site relationships were observed.

### Environmental parameter and *Campylobacter* spp. relationships

Significant relationships are highlighted in Table 4, with all Spearman Rank data presented in Supplementary Material. For both methods of detection (MPN-PCR and AS/NZS), significant (*p* < 0.05) positive correlations were observed between *Campylobacter* concentrations and that of daily rainfall, phosphorus levels, TSS and turbidity (Table 4). Relationships were also observed specifically between the *Campylobacter* concentrations obtained by the AS/NZS method, nitrogen, temperature, *E. coli* and DO, while relative humidity was the only relationship specific to results obtained by the MPN-PCR. It is interesting to note that all observed correlations, with the exception of temperature (−0.18, *P* < 0.05), were positive. The results of difference analysis (i.e., AS/NZS—MPN-PCR) indicated the potential for method bias; indeed, changes in nitrogen levels and relative humidity were correlated to the differences in *Campylobacter spp*. concentrations between the two methods.

**Table 4:** **Significant Spearman rank correlations between *Campylobacter* concentrations and environmental parameters**.

**Parameter**	**AS/NZS**	**MPN-PCR**	**Difference^a^**
Rainfall, day of sampling (mm)^(147)^	0.18	0.18	–
Phosphorus (mg/L)^(92)^	**0.40**	**0.41**	–
Nitrogen (mg/L)^(92)^	**0.30**	**–**	0.22
Total suspended sediment (mg/L)^(48)^	**0.56**	**0.43**	–
Turbidity (NTU)^(84)^	**0.51**	**0.46**	–
Max. temperature, day of (°C)^(147)^	−0.18	–	–
*E. coli* (MPN/100 mL)^(147)^	**0.22**	–	–
Dissolved oxygen (mg/L)^(52)^	0.35	–	–
Relative humidity (%)^(147)^	–	**0.22**	−0.19

*All data, from all sites, were included. Values presented have P < 0.05. Bold type indicates P < 0.01. Total number of samples in each individual analysis identified in parentheses*.

a *Results derived from Spearman correlation of subtracted values from AS/NZS and MPN-PCR*.

### *Campylobacter* spp. within-site relationships to environmental parameters

As a result of the identification of putative method bias, within-site analysis was conducted independently for MPN-PCR and AS/NZS. Significant correlative relationships are outlined in Table 5. All Spearman Rank data are presented in Supplementary Material. Relationships at Abbotsford and Morell Bridge were only identified with AS/NZS, despite a significant relationship between the methods still being maintained (σ = 0.72, *P* < 0.001). Gardiners Ck had the largest number of observed significant results with rainfall (day of), EC, temperature (day of), *E. coli* and phosphorus showing significant (*P* < 0.05) correlations irrespective of the method employed. A single relationship between *Campylobacter* concentration and humidity was observed by MPN-PCR at Kew.

**Table 5:** **Significant within site Spearman Rank correlations between *Campylobacter spp*. concentration and environmental parameters**.

	**Abbotsford**	**Morell bridge**	**Gardiners Ck**		**Kew**
	**AS/NZS**	**AS/NZS**	**MPN-PCR**	**AS/NZS**	**MPN-PCR**
Rainfall, day of sampling	–	–	**0.55^(34)^**	**0.57^(34)^**	–
Rainfall, 24 h	−0.33^(42)^	–	–	–	–
TSS	0.55^(14)^	–	–	–	–
Nitrogen	0.47^(26)^	**0.51^(27)^**	–	–	–
EC	0.42^(23)^	–	−0.57^(17)^	−**0.71^(17)^**	–
Humidity	–	–	–	0.36^(34)^	0.67^(10)^
Turbidity	–	**0.63^(25)^**	–	**0.63^(17)^**	–
Temp, day of sampling	–	–	−**0.29^(34)^**	−**0.44^(34)^**	–
Temp, 24 h	–	–	–	−**0.46^(34)^**	–
*E. coli*	–	–	**0.71^(34)^**	**0.68^(34)^**	–
Phosphorus	–	–	**0.63^(20)^**	**0.56^(20)^**	–
Flow, day of sampling	–	–	–	0.50^(34)^	–
Flow, 24 h	–	–	–	0.41^(34)^	–

*Values presented have P < 0.05. Bold type indicates P < 0.01. Total number of samples in each analysis identified in parentheses*.

## Discussion

*Campylobacter* are a major cause of gastrointestinal illness, yet, many sources of disease outbreak remain unidentified. Recreational waters (rivers, lakes and estuaries), and stormwaters which are harvested for indoor or outdoor domestic uses, can represent a significant source of infection (Koenraad et al., 1997; Moore et al., 2001; Savill et al., 2001; Sidhu et al., 2012). However, isolation and enumeration from aquatic environments can be difficult due to a multitude of environmental, biological and biophysical variables (Khan et al., 2009). The current study aimed to evaluate the intra-laboratory reproducibility of a novel DNA-extraction free MPN-PCR as an alternative to the current Australian Standard (AS/NZS) method. To undertake the evaluation three main factors were taken into consideration.

### Inter-laboratory reproducibility of culture-based methods

Due to a dearth of data from multicenter studies, on *Campylobacter* enumeration standards, it was difficult to assess if the current results deviated from normal trends. Kappa analysis and Spearman correlations provided evidence of significant relationships between the two laboratories. Although significant, there was evidence that the two methods deviated, with some samples having differences of up to 690 MPN/L. However, a single study by Scotter et al. (1993), demonstrated that even with the use of three independent culture-based methods (two of which were international standards) on the same sample, inter-laboratory *Campylobacter* results correlated, at most, 42%. Thus, it can be assumed that the results of the sensitivity, specificity and diagnostic accuracy, achieved for AS/NZS during the current study, is indicative of the normal variation observed in culture-based studies.

The introduction of variability and uncertainty, even to standardized methods, has been recognized as unavoidable for complex samples matrices (Augustin and Carlier, 2006; Pan et al., 2010). However, it is important to highlight that these studies did not assess environmentally-derived samples, which have unique, independent source-related levels of uncertainty. Analytical methods for bacterial measurement within water sources utilize sub-sampling regimes as an indication of true microbial load (Ongerth, 2013). However, microbes are not evenly distributed, spatially or temporally, thus a single sample may not be representative of actual bacterial concentrations. Recovery efficiencies for low concentration microorganisms, such as *Campylobacter*, can vary dramatically depending on water quality matrices (total suspended solids (TSS) and turbidity); which limit the volume of sample that can be processed (Pickup, 1991; Rosef et al., 2001; Ongerth, 2013). During rain events, turbidity and TSS levels within the Yarra River estuary frequently exceed 100 NTU and 100 mg/L respectively (Daly et al., 2013). Consequently, the *Campylobacter* assay filtrate volumes were reduced to ≤50 mL to enable filtration which may have resulted in a concurrent reduction in recovery. The efficiency of isolation may also be affected by the presence of competing organisms; the concentration of which have been shown to increase with filtrate volume (Rosef et al., 2001; Abulreesh et al., 2005). To date, only a single study has attempted to quantify some of the factors effecting inter-laboratory reproducibility of cultural isolation of *Campylobacter* from water sources (Khan et al., 2009). The researchers found that the low concentration of *Campylobacter* within water samples, as well as culture based method applied, may introduced a further level of variability between the sub-samples (Khan et al., 2009); as was observed between Lab-Res and Lab-Comm. However, unlike the current study, (Khan et al., 2009) did not account for the role of exogenous environmental factors in the introduction of variability; which is unique to this study.

It is recognized that irrespective of introduced uncertainties Lab-Res still retained higher detected concentrations of *Campylobacter spp*. in 68% of samples. A study by Augustin and Carlier (2006) identified factors including resuscitation technique, method of plating, presence of inhibitors (chemical and biological) as well as mode and manufacturer of culture media effected inter-laboratory reproducibility of culture-based methods. Augustin and Carlier (2006) also suggested culture media (source and preparation) was a key factor in observed count differences between laboratories, and may account for some of the differences observed in the current study. Furthermore, previous studies (Williams et al., 2012) have also identified a possible culture-associated bias toward certain *Campylobacter* species. A concurrent study within the Lab-Res has identified *Campylobacter coli* as the predominant species within the Yarra River estuary (data not shown). Differences in the observed inter-laboratory concentrations could represent culture-associated bias, with one laboratory able to cultivate a subset of Campylobacters that cannot, for reasons yet to be defined, be isolated within the other facility. A further investigation of this hypothesis is currently underway. However, in combination, the analysis suggests that low diagnostic accuracy between facilities is not a unique phenomenon, with small alterations in technical aspects having large impacts on final results.

### Intra-laboratory evaluation of MPN-PCR and AS/NZS

The current study applied ISO 22174:2005 parameters to evaluate the diagnostic potential of the proposed MPN-PCR assay (Hoorfar and Cook, 2003; Standarisation ISO, 2005). The standard, which summarizes the application of PCR based technologies for diagnosis of food-borne pathogens, has been previously applied to *Campylobacter* enumeration from food (Josefsen et al., 2004a,b,c).

ISO 22174:2005 outlines that for proposed PCR assays, pre-enrichment procedures should be equivalent to a culture-based standard to enable easy implementation into routine laboratory practices (Hoorfar and Cook, 2003; Standarisation ISO, 2005). Consequently, no variation from the outlined AS/NZS filtration and enrichment steps were undertaken. Enrichment prior to PCR enhances sensitivity by increasing the number of target cells available for amplification while reducing relative inhibitor levels (Hoorfar and Cook, 2003). Limiting the presence of inhibitory substances within complex water and soil samples is essential for accurate enumeration. However, it is important to note that *Campylobacter* culture media contains known PCR inhibitors which may affect assay outcomes (Josefsen et al., 2004c; Schrader et al., 2012). For example, Josefsen et al. (2004c) applied direct amplification from Preston enrichment culture and observed inhibition. To reduce inhibitory effects Josefsen et al. (2004c) applied a simplified DNA purification protocol. However, Josefsen et al. (2004c) did not attempt a simple dilution method, as recommended in ISO 22174:2005, which, in the current study, was found to negate any inhibitory effects introduced from the culture media.

ISO 22174:2005 also outlines that any proposed PCR assay should have a diagnostic accuracy equivalent or greater than the standard method it is replacing (Hoorfar and Cook, 2003). The results of the inter-laboratory study demonstrated a diagnostic accuracy of 76.5% for AS/NZS. However, it is important to note that calculation of the inter-laboratory diagnostic accuracy for the culture-based method used 69 samples in comparison to the 147 applied to the intra-laboratory MPN-PCR assay.

Three sub-sampling regimes were investigated for use with estuarine waters based on sensitivity of detection (to ensure enumeration of both high and low concentrations of *Campylobacter*) and ease of filtration of the turbid water samples. To date, environmental water sampling regimes, for *Campylobacter*, often advocate the use of large sample volumes (St-Pierre et al., 2009; Lévesque et al., 2011) for enhanced diagnostic accuracy. However, this limits their application to low turbidity, low TSS waters. A study by Abulreesh et al. (2005) demonstrated that for routine diagnostics, of turbid samples, filtrate volumes below 1000 mL decreased false-negative rates by limiting co-inoculation of heterotrophic bacteria; in turn, improving the diagnostic accuracy. To date, the diagnostic accuracy, sensitivity and specificity of *Campylobacter* PCR assays incorporating low volume filtrates (<200 mL), such as those applied in the current study, have not been assessed. However, it is important to recognize that this has not prevented their use in risk assessment studies (de Man et al., 2014).

Results of the current study demonstrated that for 41 of the 147 estuarine samples (i.e., for 28% of samples), the MPN-PCR method did not achieve the same *Campylobacter* concentration as AS/NZS. Interestingly, 29 of the 41 non-matching samples had a significantly higher enumerated *Campylobacter* concentration by the MPN-PCR method. Reports by other authors have also shown enhanced sensitivity of molecular methods in comparison to culture-dependent techniques (Savill et al., 2001; Josefsen et al., 2004b; Khan et al., 2009; St-Pierre et al., 2009; Bargellini et al., 2010; Lévesque et al., 2011). Suggested reasons for observed increases in sensitivity include amplification of DNA from damaged, dead or viable but non-culturable cell forms and competition by heterotrophic bacteria inhibiting *Campylobacter* culture (Augustin and Carlier, 2006; St-Pierre et al., 2009; Lévesque et al., 2011). The future inclusion of estuarine water controls, during inter-laboratory method evaluation, will aid in determining the true-effect of contaminating DNA on assay sensitivity.

The percent sensitivity is used to indicate the ability of an assay to detect a true positive within a population (Cook et al., 2007; Šimundić, 2008). In contrast, specificity measures the capacity of a method to detect a true negative (Cook et al., 2007; Šimundić, 2008). For wet weather sampling the sensitivity and specificity of the method were observed to be 95.5 and 93.3% respectively. In contrast, the sensitivity of the dry weather regime was higher (99.5%) with a lower overall specificity (87.6%). These results suggest that performance of the MPN-PCR, in its ability to detect true negatives and true positives was greatest for the smaller volume wet weather regime. The decrease of specificity, associated with an increase in false negatives, during the dry weather regime may have been associated with the application of larger filtrate volumes. The concentration of inhibitors and heterotrophic bacterial contamination, within the broth, may have increased, resulting in inhibition of the downstream PCR assay. Lending further support to this hypothesis is the observation, that for both regimes, the specificity of the assay was lowest with the highest filtered volume and improves as filtrate volumes decrease.

Likelihood ratios (LR) determine the probability of a specific test result occurring only in positive populations to that of the probability of it occurring within negative populations (Deeks and Altman, 2004; Šimundić, 2008). LR+ ratios >10 in combination with LR- ratios <0.01 are considered to provide the strongest evidence of diagnostic accuracy (Deeks and Altman, 2004; Šimundić, 2008). Irrespective of the filtration regime applied, the MPN-PCR assay displayed ratios of LR+ 9.4 and LR- 0.03, indicating that the method has strong diagnostic accuracy under most conditions tested, and higher than that of AS/NZS (LR+ 4.7 and LR- 0.37). As was observed previously, the lowest LR+ results were achieved with the largest filtrate volumes, and may be directly associated with the presence of bacterial and environmental inhibitors. In accordance with ISO 22174:2005 the proposed PCR assay has a diagnostic accuracy, sensitivity and specificity greater than the standard method when applied to complex estuarine-derived water samples (Hoorfar and Cook, 2003).

### Spatial and environmental parameters relationships

Previous studies on the Yarra River estuary have demonstrated spatial relationships between fecal indicator organisms and sampling locations (Daly et al., 2013). However, previous assessment of *Campylobacter* relationships utilized a small dataset, limited environmental data and a single method approach (data not shown). Thus, only limited assessment of the pathogen-factorial relationships could be conducted. In the current study, *Campylobacter* concentrations between two closely situated sites, Abbotsford and Dights Falls, correlated in 53% of samples by AS/NZS. The observed relationship was not unexpected with previous data (also conducted with AS/NZS) suggesting that the primary source of *Campylobacter*, into the estuary, is derived from agricultural inputs above Dights Falls (data not shown).

Estuaries are dynamic environments affected by a multitude of variables. Consequently, infectious disease transmission, within these systems, “should be viewed within an ecological framework” (Patz et al., 2003). The understanding of pathogen-environment relationships is essential for improved detection and the evaluation of persistence; both of which aid in prevention and lowering of disease rates (Schets et al., 2011a,b). To date, studies investigating parameter-bacteria relationships have primarily applied single method approaches (Rodriguez and Araujo, 2010; Rodríguez and Araujo, 2012). It is of significant concern that researchers often fail to recognize or evaluate the uncertainty introduced as a direct result of technique applied. Consequently, current cited relationships (reviewed in Sterk et al., 2013) may have been inaccurately identified, which may explain some of the observed between-study inconsistencies. To our knowledge, the current study is the first to assess method effect on the evaluation of environmental relationships and determine significant links between these and *Campylobacter*.

Rainfall, phosphorus, TSS and turbidity levels were observed to correlate with *Campylobacter* concentrations, across the estuary, irrespective of the method applied. Relationships between bacteria and these parameters have been previously reported (Gachter et al., 1988; McCarthy et al., 2012; Batabyal et al., 2014). However, the existence of these specific parameter associations has not been demonstrated for *Campylobacter spp*. within estuarine settings. At Gardeners Ck within site analysis identified relationships between the pathogen, rainfall (day of sampling), conductivity, temperature (day of sampling), *E. coli* and phosphorus levels. Interestingly, this was the only site in which parameter relationships were identified by both MPN-PCR and AS/NZS. The shallow depth, low flow and difference in stormwater inputs at Gardeners Ck may have contributed to the increased number of relationships observed; as small alterations in conditions may have a larger effect on the microbial community.

Difference analysis conducted on the total Yarra River dataset identified two possible sources of method specific bias. The data demonstrated that increases in total nitrogen (TN) resulted in a concurrent increase in *Campylobacter* detected by AS/NZS, with less difference observed between concentrations derived by the two enumeration methods. TN measures all forms of nitrogen (nitrate, nitrite and ammonia) within environmental water samples. Increased concentrations of total nitrogen have been shown to support and enhance the growth of fecal indicators in a range of environments (Hirn et al., 1980; Wittman et al., 2013; Cederlund et al., 2014). *In vitro, Campylobacter* survival has been demonstrated to be supported by the addition of nitrate to selective agars (Sellars et al., 2002; Pittman et al., 2007). It is therefore hypothesized that the presence of exogenous nitrogen, and in particular nitrate, carried on the filter and into the enrichment culture, further promoted *Campylobacter* growth under the described experimental conditions. In contrast, it was observed that as relative % humidity increased the concentration of *Campylobacter* derived by MPN-PCR differed more significantly from those of AS/NZS; MPN-PCR having the higher detected bacterial concentration. The specific effect of humidity on the growth of *Campylobacter* within enrichment cultures remains unknown and requires further evaluation. However, previous studies have demonstrated that high humidity supports the growth of a range of non-pathogenic bacteria (Arundel et al., 1986). Increased competition from co-inoculated, endogenous bacteria would result in decreased isolation of *Campylobacter* by the culture-based method but would have limited effect on the detection of specific DNA by MPN-PCR.

In order to provide adequate assessment of risks, we must understand within-lab and between lab uncertainties. We must also focus on developing faster, cheaper and more accurate tools for quantifying potential health hazards. We contribute to the development of faster, more accurate measurement of *Campylobacter* levels in urban water systems. The MPN-PCR method presented has improved diagnostic accuracy, specificity and sensitivity in comparison to AS/NZS and is a fraction of the lab and consumable costs and time. The MPN-PCR approach may also represent a viable alternative to other culture-based international standard procedures for *Campylobacter* isolation, such as ISO 17995:2005. Inter-laboratory investigations will further define the diagnostic performance for recreational waters.

Environmental parameter relationship information is essential for accurate hazard identification, mitigation and the calculation of exposure dose response. The application of a dual-method approach to *Campylobacter* enumeration allowed method specific effects on the identification of environment-pathogen relationships to be evaluated. The results identified the potential for method-specific bias and introduced uncertainty. Further application of dual-method approaches, such as the one implemented in this study, are required to define the total effect of method introduced-bias on evaluation of pathogen-environment interactions.

### Conflict of interest statement

The authors declare that the research was conducted in the absence of any commercial or financial relationships that could be construed as a potential conflict of interest.
